# Dual-trajectory of TyG levels and lifestyle scores and their associations with ischemic stroke in a non-diabetic population: a cohort study

**DOI:** 10.1186/s12933-024-02313-z

**Published:** 2024-06-28

**Authors:** Hui Zhou, Xiong Ding, Yulong Lan, Wei Fang, Xiaojie Yuan, Yan Tian, Shuohua Chen, Shouling Wu, Dan Wu

**Affiliations:** 1 Xiangya School of Nursing, Central South University, Changsha, China; 2 School of Public Health, Wuhan University, Wuhan, China; 3 Global Health Research Center, Duke Kunshan University, Suzhou, China; 4 The Second Affiliated Hospital of Shantou University Medical College, Shantou, China; 5 Centre for Precision Health, School of Medical and Health Sciences, Edith Cowan University, Joondalup, WA Australia; 6 Department of Cardiology, Second Affiliated Hospital of Air Force Medical University, Xi’an, China; 7 Department of Epidemiology, Ministry of Education Key Lab of Hazard Assessment and Control in Special Operational Environment, School of Public Health, Air Force Medical University, Xi’an, China; 8 School of Public Health, North China University of Science and Technology, Tangshan, China; 9 Department of Cardiology, Kailuan General Hospital, 57 Xinhua East Rd, Tangshan, China; 10 Department of Endocrinology, The First Affiliated Hospital of Shantou University Medical College, Shantou, China

**Keywords:** Cohort study, Triglyceride-glucose index, Lifestyle, Dual-trajectory, Ischemic stroke

## Abstract

**Background:**

The Triglyceride-glucose (TyG) index, a surrogate marker of insulin resistance, has been implicated in the risk of ischemic stroke. However, the interplay between TyG levels, lifestyle factors, and their collective impact on stroke risk in non-diabetic populations remains inadequately explored. This study aims to evaluate the association of ischemic stroke with the joint development of the TyG index and lifestyle in the non-diabetic population.

**Methods:**

In this prospective cohort study, data was collected across three consecutive biennial surveys of the Kailuan Study from 2006 to 2011. The dual-trajectory model was used to determine the temporal development of TyG levels and lifestyle scores. Statistical analysis involved Cox regression models to evaluate the association between TyG-lifestyle trajectories and ischemic stroke risk, adjusting for potential confounders.

**Results:**

A total of 44,403 participants were included, with five distinct TyG levels and lifestyle scores trajectory subtypes identified. In the multivariable-adjusted analyses, significant differences in ischemic stroke risk among the trajectory subtypes. Group 5, characterized by the highest TyG levels and moderate lifestyle scores, exhibited the greatest ischemic stroke risk (HR = 1.81, 95% CI: 1.51–2.18), while group 4, with moderate TyG levels and higher lifestyle scores, demonstrated the lowest risk (HR = 1.19, 95% CI: 1.04–1.37), compared with group 3. Participants with elevated TyG levels were at an increased risk of ischemic stroke in cases of pronounced insulin resistance, even with a healthy lifestyle.

**Conclusions:**

This study reveals the significant associations between the identified TyG and lifestyle trajectories and the stratification of ischemic stroke risk among non-diabetics. The TyG index is a valuable indicator for assessing insulin resistance. However, the potential benefits of lifestyle changes for those with significantly high TyG levels need to be clarified by more research to develop more effective stroke prevention strategies.

**Supplementary Information:**

The online version contains supplementary material available at 10.1186/s12933-024-02313-z.

## Introduction

Stroke, particularly ischemic stroke, remains a leading cause of mortality and morbidity, posing a substantial healthcare challenge, with notably rising rates in regions like China [1, 2]. The triglyceride-glucose (TyG) index, derived from fasting triglyceride and blood glucose levels, has recently been recognized as a valuable clinical marker indicative of insulin resistance and disease progression [3]. This index is also increasingly associated with the risk of ischemic stroke [4]. Concurrently, extensive research highlights the critical influence of lifestyle factors on the risk of ischemic stroke. While variations in the TyG index and lifestyle factors over time have been documented, showing that both the extent and duration of exposure to specific TyG levels and lifestyle patterns can increase stroke risk, a comprehensive analysis of their dynamic trajectories and combined effects remains underexplored in contemporary research.

There is significant variability in TyG levels and lifestyle changes throughout an individual’s life, characterized by a pronounced correlation between them. Although several studies have documented the patterns of TyG and lifestyle trajectories separately, few have integrated both factors to elucidate the observed heterogeneity. Our previous research in a general population cohort identified five distinct trajectory subtypes for TyG levels and lifestyle scores, revealing their associations with stroke risk [5]. Furthermore, we observed that the predictive ability of the TyG index for cardiovascular disease (CVD) incidence was modulated by blood glucose levels, with notable differences in CVD incidence across these subgroups being significant exclusively in the non-diabetic population [5]. Conversely, Zhao et al. reported no significant link between high TyG index and ischemic stroke risk following hospital discharge in non-diabetic patients with non-ST-segment elevation acute coronary syndrome [6]. These contrasting results highlight the need for a detailed examination of the intertwined evolution of TyG levels and lifestyle factors, especially their combined influence on the risk of ischemic stroke in non-diabetics.

To our knowledge, no study has yet described and explored the impact on ischemic stroke based on the co-evolutionary pattern of the TyG index and lifestyle. Hence, this study for the first time provided data to characterize the dual-trajectory evolution of TyG levels and lifestyle in a non-diabetic population, evaluating the joint effect on the risk of ischemic stroke, thereby informing more precise and efficacious preventive strategies.

## Methods

### Study population and study design

**Fig. 1:**
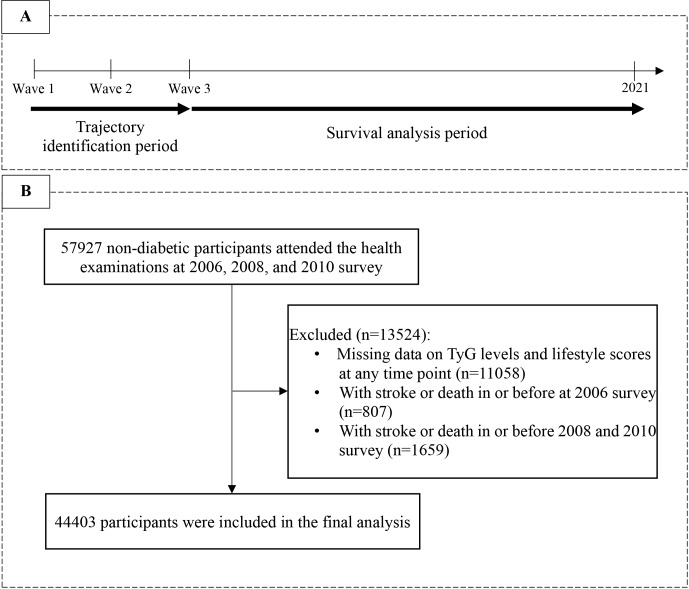
**A** Study design for examining the associations between dual-trajectory and ischemic stroke risk; **B** Flow chart for the selection of study participants

Data for this study were obtained from the Kailuan Study, a comprehensive health check-up project involving more than 100 thousand individuals focused on the investigation and intervention of CVD risk factors. Detailed information about this project has been previously described [7]. Initiated in 2006, the study has undergone biennial follow-ups, with data collection consisting of questionnaires, physical examinations, and laboratory assessments. In this study, we included active and retired workers who participated in all waves, spanning from wave 1 (2006) to wave 3 (2010, the baseline for this study) of the health check-ups and did not have diabetes. An exploration of the long-term dual-trajectory of TyG levels and lifestyle was conducted, designating 2006–2011 as the “trajectory identification period” and 2012–2021 as the “survival analysis period.” Exclusions were made for participants who lacked complete data on triglycerides, fasting blood glucose, and lifestyle domains at or before the baseline wave (*n* = 11,058), as well as those who died, or experienced a stroke during the trajectory identification period (*n* = 2466) to ensure that all participants were free from ischemic stroke upon commencement of the “survival analysis period.” Ultimately, 44,403 participants were included in the final analysis. Figure 1 provides a flow chart that depicts the participant selection process for the present study.

This study was conducted following the guidelines outlined in the Declaration of Helsinki and was approved by the Ethics Committee of Kailuan General Hospital (Approval Number: 2006–05). Written informed consent was obtained from all participants.

### Assessment of the TyG index and lifestyle

The TyG index was calculated using the formula Ln [fasting triglycerides (mg/dL) × fasting blood glucose (mg/dL)/2] [8]. Measurements for fasting blood glucose and fasting triglycerides were conducted following standard laboratory procedures at Kailuan General Hospital. Lifestyle was assessed using a scoring system encompassing five dimensions: smoking habits, alcohol consumption, physical activity, sedentary behavior, and salt intake [9]. Trained personnel gathered information on each aspect through a standardized questionnaire, assigning scores on a three-point scale ranging from 0 to 2. Cumulative scores ranged from 0 (lowest) to 10 (highest). Smoking habits and alcohol consumption were categorized as never, current or past. Sedentary behavior and physical activity were classified as poor, intermediate, or ideal. Salt intake, a proxy for dietary quality, was assessed through a questionnaire. Participants’ salt intake levels were categorized as follows: ≥ 10 g/d, 6–9 g/d, and < 6 g/d. These specific categorizations were employed as they align with established dietary guidelines [10]. Diabetes mellitus was defined as self-reported diabetes, use of glucose-lowering drugs, or fasting blood glucose ≥ 7 mmol/L (Additional file 1: Table S1).

### Data collection and definition

Covariate data were obtained through a combination of questionnaires, anthropometric measurements, and laboratory assessments. Demographic information, such as age, gender, marital status, and educational background, was collected. Medical history and medication usage were evaluated through self-reports and reviews of medical records. Hypertension was defined as systolic blood pressure (SBP) ≥ 140 mmHg or diastolic blood pressure (DBP) ≥ 90 mmHg, use of antihypertensive drugs, or a self-reported history of physician-diagnosed hypertension. Trained staff measured weight, height, SBP, and DBP. Body mass index (BMI) was calculated by dividing weight in kilograms by the square of height in meters [11]. The estimated glomerular filtration rate (eGFR) was computed using the Chronic Kidney Disease Epidemiology Collaboration creatinine equation [12].

Blood samples were drawn from the cephalic vein in the morning after an overnight fast (> 8 h). All biochemical parameters, including high-density lipoprotein cholesterol (HDL-C), low-density lipoprotein cholesterol (LDL-C), high-sensitivity C-reactive protein (s-CRP), serum fasting triglycerides, fasting blood glucose, and serum creatinine, were measured on the Hitachi 7600 auto-analyzer (Hitachi, Tokyo, Japan).

### Outcomes

The outcome of this study was the incidence of ischemic stroke. Diagnostic codes followed the International Classification of Diseases - Tenth Revision, I63. A panel of experts annually reviewed discharge records from 11 local hospitals and a database linked to the municipal social insurance agency to identify patients with suspected ischemic stroke. The diagnosis of ischemic stroke adhered to World Health Organization criteria, involving neurological signs, symptoms, and imaging examinations (e.g., computed tomography or magnetic resonance imaging) [13]. Participants were prospectively followed from the baseline year until the occurrence of ischemic stroke, death, or the end of the follow-up period on December 31, 2021, whichever came first.

### Statistical analysis

All analyses were conducted using SAS version 9.4 (SAS Institute Inc., Cary, NC), and statistical significance was set at a two-sided *P* < 0.05. Descriptive statistics were utilized to summarize the demographic and clinical characteristics of participants. Continuous variables were presented as mean ± standard deviation, while categorical variables were expressed as percentages.

#### Phase 1: Identification of TyG levels and lifestyle score trajectory groups

We employed a group-based dual-trajectory model to identify the temporal progression of TyG levels and lifestyle scores, utilizing a semi-parametric approach. This method allows to examination of the dynamics of both indicators simultaneously, and the result suggests the possibility that the two indicators may be linked by a shared underlying etiological process. Specifically, it assesses the likelihood of lifestyle score trajectories under conditions in which individuals follow a specific TyG-level trajectory, without presupposing a premise. Following Nagin’s recommendations [14], we employed a two-stage model selection process to identify the best-fit model. In the first stage, we determined the number of groups to include in the model, ranging from two to six clusters. In the second stage, we adjusted the order of the trajectory polynomials, specifying the shape of each trajectory (linear, quadratic, or cubic). Time was measured in years since 2006, and we used data from three waves of TyG levels and lifestyle scores to estimate the trajectories. Model fitting was conducted iteratively by comparing models with two to six groups and trajectory shapes. The optimal dual-trajectory model for TyG levels and lifestyle scores was selected based on several evaluation metrics, including the smallest Bayesian Information Criterion (BIC) value, smallest Akaike’s Information Criterion (AIC) value, smallest log-likelihood value, and maximum Entropy. Additionally, we assessed the average posterior probability (above 0.7) and the predicted probability of group membership (above 5%) for further validation [15].

#### Phase 2: Association analysis between dual-trajectory and ischemic stroke

Person-years were calculated from the 2010 survey date until the first occurrence of ischemic stroke, death, or the end of follow-up on December 31, 2021, whichever came first. We estimated ischemic stroke probabilities using the Kaplan-Meier method and compared inter-group differences using log-rank tests. The model met proportional assumption criteria per Schoenfeld residuals and log-log tests. Cox proportional hazards regression was employed to explore the associations between subtypes of TyG levels and lifestyle scores trajectories and the risk of ischemic stroke, using Group 3 (representing the lowest TyG levels and the highest lifestyle scores) as the reference group. Hazard ratios (HRs) and 95% confidence intervals (CIs) were reported after adjusting confounders consisting of age, sex, marital status, education background, BMI, LDL-C, HDL-C, ln hs-CRP, ln eGFR, hypertension, use of antihypertensive and hypolipidemic medications. We also conducted stratified analyses by age, sex, BMI, hypertension, and hyperlipidemia to investigate the potential impact of dual-trajectory subtypes of TyG and lifestyle on ischemic stroke risk. Likelihood ratio tests evaluated interactions between stratified variables and dual-trajectory. Additionally, to test the robustness of our findings, several sensitivity analyses were performed, such as adjustments for baseline conditions, exclusion of participants on medications, exclusion of those with inflammation (hs-CRP ≥ 10 mg/L), exclusion of ischemic stroke events within the initial 2 years of follow-up, and application of a Fine-Gray competing risk model treating deaths as competing events.

## Results

### Baseline characteristics

**Fig. 2:**
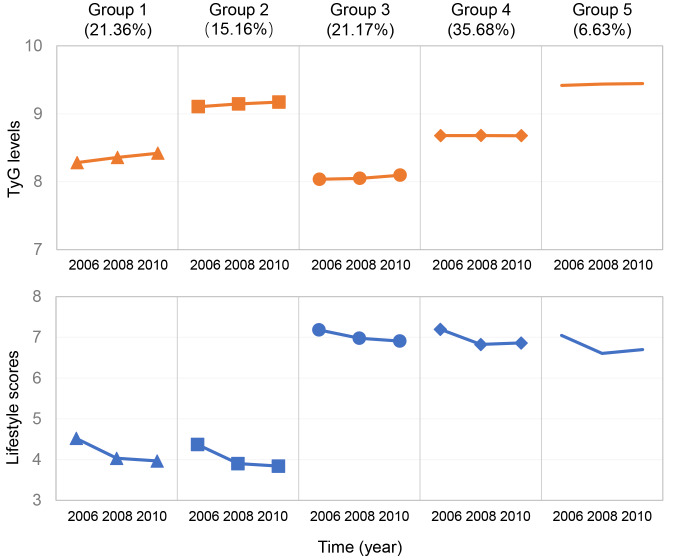
Dual-trajectory groups of TyG levels and lifestyle scores using group-based dual-trajectory modeling. Note: Dots show group-specific mean observed levels while solid lines represent the best fitted trajectories. TyG index and lifestyle scores were modeled as a function of follow-up time. Group 1 = lower TyG levels and lower lifestyle scores; group 2 = higher TyG levels and the lowest lifestyle scores; group 3 = the lowest TyG levels and the highest lifestyle scores; group 4 = moderate TyG levels and higher lifestyle scores; and group 5 = the highest TyG levels and moderate lifestyle scores

A total of 44,403 non-diabetic participants, with a mean age of 52.42 ± 11.77 years, were included in this study. We identified five distinct subtypes of dual-trajectory, denoted as group 1 (lower TyG levels and lower lifestyle scores, *n* = 9485, 21.36%), group 2 (higher TyG levels and the lowest lifestyle scores, *n* = 6730, 15.16%), group 3 (the lowest TyG levels and the highest lifestyle scores, *n* = 9399, 21.17%), group 4 (moderate TyG levels and higher lifestyle scores, *n* = 15,842, 35.68%), and group 5 (the highest TyG levels and moderate lifestyle scores, *n* = 2947, 6.63%), as detailed in Fig. 2. The average posterior probability of these five subtypes was 0.84 (Additional file 1: Table S2). Table 1 presents the baseline characteristics of non-diabetic participants grouped by TyG levels and lifestyle score trajectories. Participants in group 5, when compared to other groups, were more likely to be older, had hypertension and dyslipidemia, using antihypertensive and hypolipidemic medications, and had higher BMI, SBP, DBP, LDL-C, and hs-CRP levels.

**Table 1:** Basic characteristics of participants

Characteristics	Total	Group 1	Group 2	Group 3	Group 4	Group 5
N	44,403	9485	6730	9399	15,842	2947
Age, year	52.21 ± 11.84	49.71 ± 11.26	48.35 ± 9.69	53.03 ± 12.93	54.42 ± 11.84	54.61 ± 10.72
Sex						
Female	10,664 (24.02)	175 (1.85)	52 (0.77)	4182 (44.49)	5434 (34.30)	821 (27.86)
Male	33,739 (75.98)	9310 (98.15)	6678 (99.23)	5217 (55.51)	10,408 (65.70)	2126 (72.14)
Marital status						
Others	569 (1.28)	149 (1.57)	72 (1.07)	157 (1.67)	160 (1.01)	31 (1.05)
Married or remarriage	43,834 (98.72)	9336 (98.43)	6658 (98.93)	9242 (98.33)	15,682 (98.99)	2916 (98.95)
Education background						
Illiterate/elementary	2870 (6.46)	624 (6.58)	382 (5.68)	573 (6.10)	1091 (6.89)	200 (6.79)
Middle school or above	41,533 (93.54)	8861 (93.42)	6348 (94.32)	8826 (93.90)	14,751 (93.11)	2747 (93.21)
BMI, kg/m^2^	24.97 ± 3.27	24.18 ± 3.02	26.10 ± 3.04	23.69 ± 3.13	25.37 ± 3.22	26.76 ± 3.09
SBP, mm Hg	129.64 ± 18.32	127.45 ± 16.72	131.78 ± 16.72	125.21 ± 18.90	131.45 ± 18.77	136.28 ± 18.33
DBP, mm Hg	84.03 ± 10.50	83.50 ± 10.04	87.02 ± 10.21	80.60 ± 10.35	84.52 ± 10.35	87.26 ± 10.56
HDL-C, mmol/L	1.56 ± 0.42	1.62 ± 0.44	1.50 ± 0.40	1.69 ± 0.44	1.50 ± 0.38	1.36 ± 0.37
LDL-C, mmol/L	2.62 ± 0.73	2.58 ± 0.66	2.75 ± 0.70	2.38 ± 0.71	2.71 ± 0.73	2.75 ± 0.83
hs-CRP, mg/L	2.01 ± 3.00	1.89 ± 2.94	2.19 ± 3.14	1.87 ± 2.87	1.97 ± 2.97	2.59 ± 3.36
eGFR, ml/min/1.73 m^3^	90.62 ± 18.37	96.97 ± 16.25	96.11 ± 16.35	89.98 ± 19.03	85.71 ± 18.32	86.09 ± 17.81
Hypertension	20,349 (54.17)	3932 (41.45)	3827 (56.86)	3068 (32.64)	7732 (48.81)	1790 (60.74)
Hyperlipidemia	14,750 (33.22)	2151 (22.68)	4221 (62.72)	1253 (13.33)	4817 (30.41)	2308 (78.32)
Medication usage						
Antihypertensive	4313 (9.71)	788 (8.31)	902 (13.40)	518 (5.51)	1669 (10.54)	436 (14.79)
Hypolipidemic	273 (0.61)	37 (0.39)	75 (1.11)	17 (0.18)	97 (0.61)	47 (1.59)
TyG						
2006/07_TyG	8.57 ± 0.60	8.27 ± 0.40	9.13 ± 0.48	8.00 ± 0.34	8.69 ± 0.42	9.47 ± 0.45
2008/09_TyG	8.60 ± 0.59	8.35 ± 0.40	9.16 ± 0.46	8.01 ± 0.35	8.69 ± 0.37	9.50 ± 0.43
2010/11_TyG	8.63 ± 0.58	8.41 ± 0.40	9.19 ± 0.47	8.06 ± 0.34	8.69 ± 0.38	9.50 ± 0.45
Lifestyle						
2006/07_Lifestyle	6.19 ± 2.03	4.47 ± 1.72	4.34 ± 1.76	7.20 ± 1.36	7.24 ± 1.38	7.11 ± 1.43
2008/09_Lifestyle	5.81 ± 2.11	3.98 ± 1.48	3.87 ± 1.51	7.02 ± 1.47	6.84 ± 1.64	6.67 ± 1.70
2010/11_Lifestyle	5.79 ± 2.07	3.90 ± 1.62	3.80 ± 1.60	6.95 ± 1.30	6.89 ± 1.38	6.77 ± 1.46

BMI, body mass index; DBP, diastolic blood pressure; eGFR, estimated glomerular filtration rate; HDL-C, high-density lipoprotein cholesterol; hs-CRP, high sensitivity C-reactive protein; LDL-C, low-density lipoprotein cholesterol; SBP, systolic blood pressure; TyG, triglyceride-glucose

### Dual-trajectory subtypes of TyG levels and lifestyle scores and ischemic stroke

**Fig. 3:**
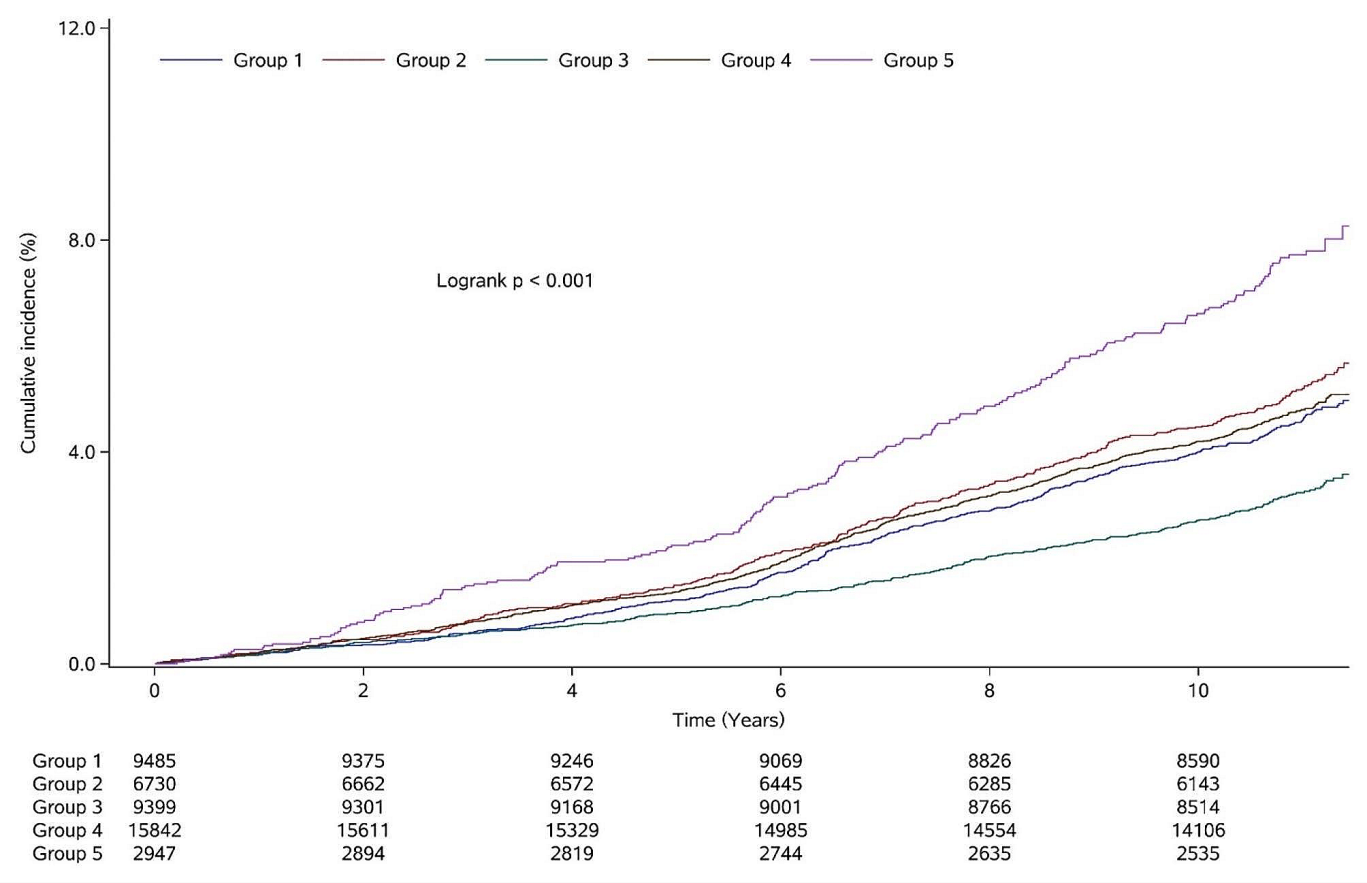
The Kaplan–Meier curves of ischemic stroke. Note: group 1 = lower TyG levels and lower lifestyle scores; group 2 = higher TyG levels and the lowest lifestyle scores; group 3 = the lowest TyG levels and the highest lifestyle scores; group 4 = moderate TyG levels and higher lifestyle scores; and group 5 = the highest TyG levels and moderate lifestyle scores

Over a median follow-up period of 10.47 (10.58–11.00) years, a total of 2012 participants experienced ischemic stroke. Notably, the group characterized by the highest TyG levels and moderate lifestyle scores (group 5) exhibited the highest incidence rate of ischemic stroke (7.20 per 1000 person-years), while participants in group 3, characterized by the lowest TyG levels and the highest lifestyle scores, had the lowest incidence rate (2.97 per 1000 person-years). Kaplan-Meier curves demonstrated a significantly higher risk of ischemic stroke in group 5 compared to other groups (*P* < 0.001 for a log-rank test; Fig. 3).

After adjusting for potential confounders (Table 2), participants in group 5, characterized by the highest TyG levels and moderate lifestyle scores, exhibited the highest ischemic stroke risk (HR = 1.81, 95% CI: 1.51–2.18). Following this, group 2, with higher TyG levels and the lowest lifestyle scores, also displayed an elevated risk (HR = 1.47, 95% CI: 1.24–1.74), as did group 1, characterized by lower TyG levels and lower lifestyle scores (HR = 1.40, 95% CI: 1.20–1.64). Conversely, participants in group 4, with moderate TyG levels and higher lifestyle scores, had the lowest risk of ischemic stroke (HR = 1.19, 95% CI: 1.04–1.37).

**Table 2:** Incidence of ischemic stroke according to dual-trajectory of TyG levels and lifestyle scores

	Dual-trajectory of TyG levels and lifestyle scores, HR (95% CI)				
	Group 1	Group 2	Group 3	Group 4	Group 5
Ischemic stroke					
Event/total	428/9485	346/6730	292/9399	729/15,842	217/2947
Incidence rate*	4.28 (3.89–4.70)	4.86 (4.37–5.39)	2.97 (2.64–3.33)	4.43 (4.12–4.76)	7.20 (6.30–8.22)
Model 1	1.54 (1.32–1.79)	1.94 (1.65–2.28)	Reference	1.44 (1.26–1.65)	2.39 (2.00-2.85)
Model 2	1.51 (1.30–1.77)	1.75 (1.49–2.07)	Reference	1.34 (1.17–1.54)	2.10 (1.75–2.51)
Model 3	1.40 (1.20–1.64)	1.47 (1.24–1.74)	Reference	1.19 (1.04–1.37)	1.81 (1.51–2.18)

*Cases per 1000 person-years

Model 1 adjusted for age and sex

Model 2 included covariates in model 1 and marital status, education background, BMI

Model 3 included covariates in model 2 and LDL-C, HDL-C, ln hs-CRP, ln eGFR, hypertension, use of antihypertensive and hypolipidemic medications

**Fig. 4:**
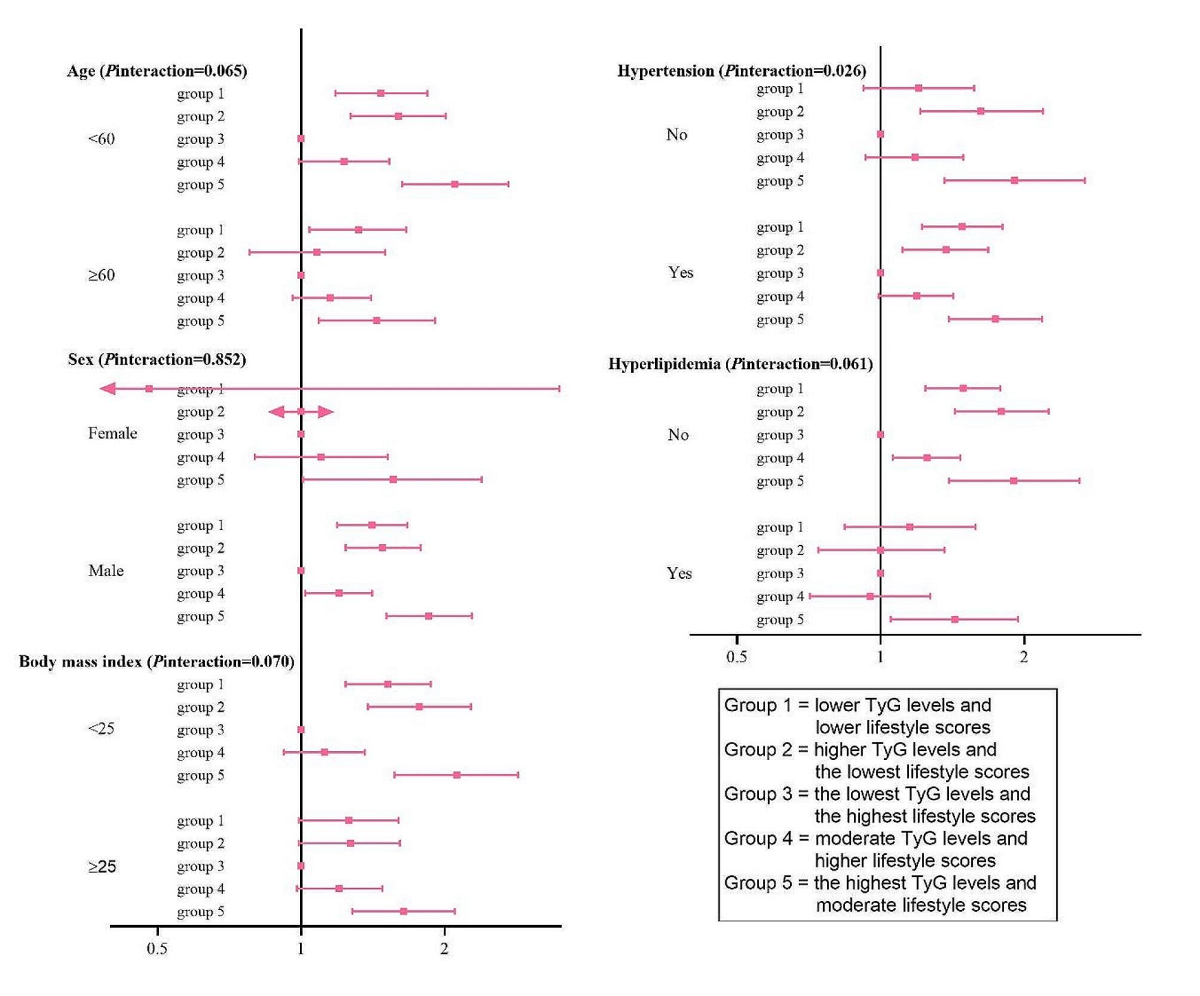
Stratified analysis of the association between dual-trajectory of TyG levels and lifestyle scores and ischemic stroke. Note: Model adjusted for age, sex, marital status, education background, body mass index, LDL-C, HDL-C, ln hs-CRP, ln eGFR, hypertension, use of antihypertensive and hypolipidemic medications

To explore the association between the dual-trajectory subtypes and ischemic stroke incidence in detail, analyses stratified by sex, age, BMI, hypertension, and hyperlipidemia were performed. The association between dual-trajectory subtypes and ischemic stroke risk showed consistency and stability across stratified variables, as demonstrated by the fact that group 5 was considered to be the most prone to ischemic stroke, whereas group 4 had a moderate TyG level but was at the lowest risk due to its favorable lifestyle. Stratification analysis also demonstrated the dual-trajectory of the TyG index and lifestyle exhibited a significant association with a higher risk of ischemic stroke in the subgroups of participants younger than 60 years, male, BMI < 25 kg/m^2^, and without hyperlipidemia (all *P* for interaction > 0.05). Interestingly, the dual-trajectory subtypes appeared to exhibit a more pronounced predicting value among patients without previous hypertension (*P* for interaction = 0.026, Fig. 4).

Furthermore, several sensitivity analyses were conducted to assess the robustness and reliability of the main results. First, adjusting for TyG levels and lifestyle scores at baseline did not substantially alter the associations (Additional file 1: Table S3). Then, the results of the analysis, including the exclusion of medication usage (Additional file 1: Table S4), exclusion of hs-CRP ≥ 10 mg/L (Additional file 1: Table S5), exclusion of outcomes within the initial 2 years of follow-up (Additional file 1: Table S6), and treating deaths as competing risk events (Additional file 1: Table S7), were consistent with the main results.

## Discussion

Using a prospective large-scale cohort, our study revealed the co-evolutionary patterns of the TyG index and lifestyle over time and evaluated their impact on the risk of ischemic stroke among the non-diabetic population in North China. Overall, we identified five distinct subtypes based on their temporal development of TyG levels and lifestyle scores. Notably, only 21.17% of participants were categorized as the type with the lowest TyG levels and the highest lifestyle scores, while a more common pattern emerged of moderate TyG levels paired with higher lifestyle scores. Our trajectory analysis showed that ischemic stroke risk varied across subtypes; those with the highest TyG levels and moderate lifestyle scores had the greatest increased risk of ischemic stroke compared with those with the lowest TyG levels and the highest lifestyle scores.

Our research contributed to the growing body of evidence on the dynamic interplay between TyG index and ischemic stroke risk, particularly in non-diabetic populations. The results of diabetes subgroup analysis in previous studies showed that the relationship between the TyG index and the risk of CVD was not stable. Some cohort studies revealed both prolonged high TyG levels [16] and significant fluctuations [17] were associated with elevated ischemic stroke risk. Another research paralleling our approach found that consistently high TyG levels increased the ischemic stroke risk in normal-weight, non-diabetic adults [18], emphasizing the necessity for meticulous monitoring and strategic management of TyG index trajectories to mitigate ischemic stroke risk. However, Zhao et al. did not find a significant association between high baseline TyG levels and the risk of ischemic stroke in non-diabetic patients with non-ST-segment elevation acute coronary syndrome [6]. Also, Jiao et al. observed the predictive value of the TyG index only among the population with diabetes [19]. Our study added the importance of methodological rigor in epidemiological research. The dynamic assessment of TyG levels and lifestyle scores through repeated measurements offers a comprehensive perspective that single-point analyses cannot provide. Furthermore, comparative analysis with existing studies reveals the distinct advantages of our dual-trajectory model. The association of TyG levels with ischemic stroke risk was previously explored independently, whereas our study method allowed for a more integrated examination. This approach not only aligned with but also extended the findings from previous studies, by illuminating the compounded effect of TyG levels and lifestyle scores on stroke risk.

In our study, we observed significant variability in ischemic stroke risk across different combinations of TyG levels and lifestyle score trajectories. Specifically, individuals in group 5 (exhibiting the highest TyG levels and moderate lifestyle scores) were at the highest risk of ischemic stroke, whereas group 4, which exhibited moderate TyG levels but higher lifestyle scores, demonstrated the lowest risk. This variance underscored the presence of distinct stroke risk profiles within the population, emphasizing the necessity of incorporating both TyG level and lifestyle score in crafting preventive strategies and tailored treatment plans. Further analysis over three-time points revealed that the average TyG levels were highest in group 5 at 9.47 mg/dL, with group 4 achieving the best lifestyle score of 7.24 points. This indicates that individuals with moderate TyG levels yet high lifestyle scores, such as those in group 4, can significantly mitigate their stroke risk through substantial lifestyle enhancements. However, high TyG levels could not be offset merely by lifestyle adjustments [5]. For the Chinese demographic, established TyG thresholds (> 8.81 mg/dL for males and > 8.73 mg/dL for females) suggest the prevalence of insulin resistance [20]. In these instances, a broader range of interventions, including medications for metabolic dysfunction and pharmacotherapy [21, 22], might be essential for effective stroke risk management.

The associations between TyG index, lifestyle, and ischemic stroke are complex and our investigation sheds light on potential mechanisms underpinning these associations. The brain is a critical target organ for insulin. When the body’s sensitivity to insulin is reduced, i.e. insulin resistance occurs, serum glucose and insulin levels are altered, leading to pathological changes such as metabolic abnormalities and cerebrovascular damage [23]. The TyG index, serving as a surrogate marker for insulin resistance, has been validated by epidemiological studies for its effectiveness [24] and its association with increased markers of atherosclerosis like carotid plaque [25] and intracranial arterial remodeling [26]. This evidence is further bolstered by the fact that metabolic dysregulations induced by insulin resistance, including elevated blood glucose [27] and lipid abnormalities [28], are recognized risk factors for atherosclerosis. Additionally, insulin resistance is linked to a systemic inflammatory state [29] and increased oxidative stress [30], both of which contribute to vascular endothelial damage, thrombosis promotion, and, consequently, an elevated risk of ischemic stroke.

Lifestyle changes are key to managing insulin resistance, with evidence showing that regular exercise [31], healthy diet [32], limiting alcohol intake, avoiding smoking cigarettes [33], and reduced levels of stress [34] notably enhance insulin sensitivity. Certain drugs, like biguanides, thiazolidinediones, and GLP-1 receptor agonists, also help prevent type 2 diabetes by increasing insulin sensitivity [35], but always consult a healthcare professional before starting any medication. Effective insulin resistance management relies on a synergistic approach that combines medical nutrition, exercise, medication, weight management, and tailored treatment strategies, highlighting the importance of personalized care.

### Limitations and strengths

Firstly, the study’s reliance on self-reported lifestyle data introduces the potential for bias, a limitation inherent to such data collection methods. Secondly, our lifestyle scoring system did not include elements such as cumulative tobacco exposure, drinking patterns, dietary balance, etc., and these omissions may affect the interpretation of the study results. Thirdly, despite comprehensive adjustments for a wide range of confounders, including sociodemographic and health-related factors, the observational nature of this study means residual confounding factors may still influence the outcomes. Fourthly, the analysis did not incorporate the potential impact of detailed medication use on ischemic stroke risk, TyG levels, and lifestyle scores, which represents a gap in fully understanding these interrelations. Fifthly, focusing on employees in North China may restrict the applicability of our findings to broader populations, suggesting a need for caution in generalizing these results. At last, while our person-oriented approach offers insightful identification of latent groups, applying this method across different demographics might yield variable results, warranting careful comparison with other studies. Despite these limitations, our study also had significant strengths. Firstly, the considerable sample size and prospective design of our cohort study stand out as significant strengths, enabling robust longitudinal analysis. Secondly, this study uniquely examines the intertwined trajectories of TyG levels and lifestyle scores, providing an integrated view of their collective impact on ischemic stroke risk among non-diabetics. Moreover, employing a person-oriented approach facilitates the identification of latent groups without preconceived assumptions, offering a more nuanced understanding of individual patterns over time compared to traditional variable-based analyses. This approach significantly reduces bias from predefined categorizations, enhancing the reliability of our conclusions.

## Conclusion

This cohort study revealed the intricate interactions between the TyG index and lifestyle factors in a non-diabetic population, reinforcing the role of the TyG index as a potential biomarker for stratified risk management. Although a healthy lifestyle may mitigate the stroke risk associated with higher TyG levels, the association between TyG level management and stroke prevention in the presence of insulin resistance warrants further study.

### Electronic supplementary material

Below is the link to the electronic supplementary material.

Supplementary Material 1

## Data Availability

The data that support the findings of this study are available from the corresponding author, Shouling Wu, upon reasonable request.

## Abbreviations

TyG: Triglyceride-glucose
CVD: Cardiovascular disease
SBP: Systolic blood pressure
DBP: Diastolic blood pressure
BMI: Body mass index
eGFR: Estimated glomerular filtration rate
HDL-C: High-density lipoprotein cholesterol
LDL-C: Low-density lipoprotein cholesterol
hs-CRP: High-sensitivity C-reactive protein
HR: Hazard ratio
CI: Confidence interval
