# Supplementary material for: Dual-trajectory of TyG levels and lifestyle scores and their associations with ischemic stroke in a non-diabetic population: a cohort study
Source: Cardiovasc Diabetol. 2024 Jun 28;23:225. doi: 10.1186/s12933-024-02313-z (PMC11214241; doi:10.1186/s12933-024-02313-z)
Supplement: Supplementary file 1 — Supplementary Material 1 [file 12933_2024_2313_MOESM1_ESM.docx]

**Supplementary materials**

**Supplementary Table 1. Definitions of poor (0 Point), intermediate (1 Point), and ideal (2 Points) for each component of lifestyle domains**

**Supplementary Table 2. Parameters based on model-adequacy criteria for dual-trajectories in the best model of each group**

**Supplementary Table 3. HRs and 95% CIs for ischemic stroke after adjusting baseline TyG levels and lifestyle scores**

**Supplementary Table 4. HRs and 95% CIs for ischemic stroke after excluding medication usage**

**Supplementary Table 5. HRs and 95% CIs for ischemic stroke after excluding** **Hs-CRP≥10 mg/L**

**Supplementary Table 6. HRs and 95% CIs for ischemic stroke after excluding outcomes within the initial 2 years of follow-up**

**Supplementary Table 7. HRs and 95% CIs for ischemic stroke treating deaths as competing risk events**

**Supplementary Table 1. Definitions of poor (0 Point), intermediate (1 Point), and ideal (2 Points) for each component of lifestyle domains**

| Lifestyle score component | Poor (0 Point) | Intermediate (1 Point) | Ideal (2 Points) |
| --- | --- | --- | --- |
| Smoking | Current smoker | Past smoker | Never |
| Alcohol consumption | Current drinker | Past drinker | Never |
| Sedentary time, h/day | ≥ 8 | 4-7 | < 4 |
| Physical activity (moderate or vigorous exercise) | No physical activity | physical activity (20+ minutes per time) 1-2 times per week, during leisure time | physical activity (20+ minutes per time) 3 times per week, during leisure time |
| Diet, based on daily salt intake (g/d) | ≥ 10 | 6-9 | < 6 |

**Supplementary Table 2. Parameters based on model-adequacy criteria for dual-trajectories in the best model of each group**

| Variables | Number of groups | Order of trajectory | Proportion of individuals in groups (%) | BIC | 2∆BIC | AIC | Average posterior probability of assignment |
| --- | --- | --- | --- | --- | --- | --- | --- |
| Lifestyle scores | 2 | 1/1 | 37.83/62.17 | -387619.7 | - | -387567.5 | 0.92/0.95 |
| TyG levels | 2 | 2/1 |  |  |  |  |  |
| Lifestyle scores | 3 | 1/1/1 | 28.00/50.25/21.75 | -379327.7 | 16584.0 | -379258.1 | 0.87/0.91/0.85 |
| TyG levels | 3 | 1/1/1 |  |  |  |  |  |
| Lifestyle scores | 4 | 1/1/1/1 | 24.86/13.29/35.70/26.15 | -372852.2 | 12951.0 | -372756.4 | 0.85/0.85/0.87/0.84 |
| TyG levels | 4 | 1/2/1/1 |  |  |  |  |  |
| **Lifestyle scores** | **5** | **1/1/1/1/1** | **21.36/15.16/21.17/35.68/6.63** | **-370176.5** | **5351.4** | **-370045.9** | **0.85/0.85/0.84/0.83/0.83** |
| **TyG levels** | **5** | **1/2/2/2/2** |  |  |  |  |  |
| Lifestyle scores | 6 | 1/1/1/1/1/1 | 13.95/18.87/4.86/18.08/34.85/9.39 | -368544.9 | 3263.2 | -368401.4 | 0.81/0.80/0.83/0.83/0.81/0.81 |
| TyG levels | 6 | 1/1/2/1/2/1 |  |  |  |  |  |

Abbreviations: TyG, triglyceride-glucose; AIC, Akaike information criterion; BIC, Bayesian information criterion.

**Supplementary Table 3. HRs and 95% CIs for ischemic stroke after adjusting baseline TyG levels and lifestyle scores**

|  | Dual-trajectory of TyG levels and lifestyle scores, HR (95% CI) | | | | |
| --- | --- | --- | --- | --- | --- |
|  | Group 1 | Group 2 | Group 3 | Group 4 | Group 5 |
| CVD |  |  |  |  |  |
| Event/total | 428/9485 | 346/6730 | 292/9399 | 729/15842 | 217/2947 |
| Incidence rate* | 4.28 (3.89-4.70) | 4.86 (4.37-5.39) | 2.97 (2.64-3.33) | 4.43 (4.12-4.76) | 7.20 (6.30-8.22) |
| Model 4 | 1.32 (1.11-1.56) | 1.26 (1.01-1.56) | Reference | 1.11 (0.95-1.30) | 1.55 (1.22-1.96) |
| Model 5 | 1.25 (1.04-1.50) | 1.26 (1.01-1.58) | Reference | 1.16 (0.99-1.35) | 1.69 (1.34-2.15) |

*Cases per 1000 person-years.

Model 4 adjusted for age, sex, marital status, education background, body mass index, LDL-C, HDL-C, ln Hs-CRP, ln eGFR, hypertension, use of antihypertensive and hypolipidemic medications, health lifestyle score and TyG in 2006/07 wave

Model 5 adjusted for age, sex, marital status, education background, body mass index, LDL-C, HDL-C, ln Hs-CRP, ln eGFR, hypertension, use of antihypertensive and hypolipidemic medications, health lifestyle score and TyG in 2010/11 wave

**Supplementary Table 4. HRs and 95% CIs for ischemic stroke after excluding medication usage**

|  | Dual-trajectory of TyG levels and lifestyle scores, HR (95% CI) | | | | |
| --- | --- | --- | --- | --- | --- |
|  | Group 1 | Group 2 | Group 3 | Group 4 | Group 5 |
| Excluding antihypertensive usage | | |  |  |  |
| Event/total | 361/8697 | 263/5828 | 252/8881 | 592/14173 | 175/2511 |
| Incidence rate* | 3.91 (3.53-4.34) | 4.23 (3.75-4.78) | 2.70 (2.39-3.06) | 4.00 (3.69-4.33) | 6.78 (5.84-7.86) |
| Model | 1.45 (1.22-1.71) | 1.48 (1.22-1.78) | Reference | 1.20 (1.03-1.40) | 1.93 (1.57-2.36) |
| Excluding hypolipidemic usage | | |  |  |  |
| Event/total | 422/9448 | 338/6655 | 291/9382 | 723/15745 | 215/2900 |
| Incidence rate* | 4.23 (3.85-4.65) | 4.80 (4.31-5.33) | 2.96 (2.64-3.32) | 4.42 (4.11-4.75) | 7.25 (6.35-8.29) |
| Model | 1.39 (1.19-1.63) | 1.46 (1.23-1.73) | Reference | 1.20 (1.04-1.38) | 1.84 (1.53-2.21) |
| Excluding all medication usage | | |  |  |  |
| Event/total | 359/8680 | 259/5790 | 252/8874 | 591/14122 | 174/2485 |
| Incidence rate* | 3.90 (3.52-4.32) | 4.20 (3.71-4.74) | 2.70 (2.39-3.06) | 4.01 (3.70-4.34) | 6.81 (5.87-7.90) |
| Model | 1.44 (1.22-1.71) | 1.47 (1.21-1.77) | Reference | 1.21 (1.04-1.41) | 1.94 (1.58-2.37) |

*Cases per 1000 person-years.

Model adjusted for age, sex, marital status, education background, body mass index, LDL-C, HDL-C, ln Hs-CRP, ln eGFR, hypertension, use of antihypertensive, and hypolipidemic medications

**Supplementary Table 5. HRs and 95% CIs for ischemic stroke after excluding Hs-CRP≥10 mg/L**

|  | Dual-trajectory of TyG levels and lifestyle scores, HR (95% CI) | | | | |
| --- | --- | --- | --- | --- | --- |
|  | Group 1 | Group 2 | Group 3 | Group 4 | Group 5 |
| Event/total | 416/9241 | 334/6515 | 283/9171 | 703/15422 | 209/2832 |
| Incidence rate* | 4.26 (3.87-4.69) | 4.84 (4.35-5.39) | 2.94 (2.62-3 31) | 4.38 (4.07-4.71) | 7.21 (6.29-8.25) |
| Model | 1.41 (1.21-1.66) | 1.50 (1.26-1.78) | Reference | 1.20 (1.04-1.38) | 1.85 (1.53-2.23) |

Abbreviation: Hs-CPR, high sensitivity C-reactive protein.

*Cases per 1000 person-years.

Model adjusted for age, sex, marital status, education background, body mass index, LDL-C, HDL-C, ln Hs-CRP, ln eGFR, hypertension, use of antihypertensive, and hypolipidemic medications

**Supplemental Table 6.** HRs and 95% CIs for ischemic stroke after excluding outcomes within the initial 2 years of follow-up

|  | Dual-trajectory of TyG levels and lifestyle scores, HR (95% CI) | | | | |
| --- | --- | --- | --- | --- | --- |
|  | Group 1 | Group 2 | Group 3 | Group 4 | Group 5 |
| Event/total | 426/9407 | 341/6688 | 272/9319 | 713/15671 | 212/2912 |
| Incidence rate* | 4.26 (3.87-4.68) | 4.79 (4.31-5.32) | 2.76 (2.45-3.11) | 4.33 (4.03-4.66) | 7.04 (6.16-8.06) |
| Model | 1.48 (1.26-1.73) | 1.53 (1.28-1.82) | Reference | 1.25 (1.08-1.45) | 1.90 (1.57-2.29) |

*Cases per 1000 person-years.

Model adjusted for age, sex, marital status, education background, body mass index, LDL-C, HDL-C, ln Hs-CRP, ln eGFR, hypertension, use of antihypertensive, and hypolipidemic medications

**Supplemental Table 7. HRs and 95% CIs for ischemic stroke treating deaths as competing risk events**

|  | Dual-trajectory of TyG levels and lifestyle scores, HR (95% CI) | | | | |
| --- | --- | --- | --- | --- | --- |
|  | Group 1 | Group 2 | Group 3 | Group 4 | Group 5 |
| Event/total | 428/9485 | 346/6730 | 292/9399 | 729/15842 | 217/2947 |
| Incidence rate* | 4.28 (3.89-4.70) | 4.86 (4.37-5.39) | 2.97 (2.64-3.33) | 4.43 (4.12-4.76) | 7.20 (6.30-8.22) |
| Model | 1.40 (1.20-1.64) | 1.47 (1.24-1.74) | Reference | 1.19 (1.04-1.38) | 1.81 (1.51-2.18) |

*Cases per 1000 person-years.

Model adjusted for age, sex, marital status, education background, body mass index, LDL-C, HDL-C, ln Hs-CRP, ln eGFR, hypertension, use of antihypertensive, and hypolipidemic medications
